# Amaranth as an opportunity crop in Africa: drought–nutrient interactions and breeding perspectives for climate resilience

**DOI:** 10.3389/fpls.2026.1841633

**Published:** 2026-05-18

**Authors:** Kenneth Chukwuka Mkpuma, Githiri Mwangi, Mercy Kidaha, Aristide Carlos Houdegbe, Emerita Njiru, Alfred Adebo Ozimati, Sognigbe N’Danikou, Happiness Oselebe, Fekadu Dinssa, Mary Abukutsa-Onyango, Roland Schafleitner, Enoch G. Achigan-Dako

**Affiliations:** 1 Jomo Kenyatta University of Agriculture and Technology, Nairobi, Kenya; 2 Centre for Crop Improvement, Nutrition and Climate Change (CCINCC), Ebonyi State University, Abakaliki, Ebonyi, Nigeria; 3 Agricultural Research Council of Nigeria (ARCN), Agricultural Research House, Mabushi, Abuja, Nigeria; 4 Genetics, Biotechnology and Seed Science Unit (GBioS), Laboratory of Crop Production, Physiology and Plant Breeding, Faculty of Agricultural Sciences, University of Abomey-Calavi, Abomey-Calavi, Benin; 5 Kenya Agricultural and Livestock Research Organization (KALRO)- Katumani Research Center, Machakos, Kenya; 6 World Vegetable Center, Eastern and Southern Africa, Duluti, Arusha, Tanzania; 7 Ecole d’Horticulture et d’Aménagement des Espaces Verts, Université Nationale d’Agriculture, Kétou, Benin; 8 World Vegetable Center, Shanhua, Taiwan

**Keywords:** amaranthus, breeding, drought, nutrient-interactions, opportunity crop

## Abstract

Amaranth (*Amaranthus* spp.) is increasingly recognized as a promising opportunity crop for improving food and nutritional security in sub-Saharan Africa under climate change. This review synthesizes current knowledge on amaranth utilization in Africa, with particular emphasis on how drought stress influences nutrient dynamics, yield, and quality, and the implications for breeding sustainable leafy vegetable systems. Evidence from both controlled and field studies indicates that moderate drought can enhance the accumulation of minerals, proteins, and secondary metabolites in leaves, whereas severe stress reduces growth, yield, and nutritional stability; in contrast, grain nutritional quality remains relatively stable under moderate water deficit. These responses are underpinned by coordinated morphological, physiological, biochemical, and molecular mechanisms, including osmotic adjustment, antioxidant defense, ABA-mediated gene regulation, and genotype-specific accumulation of protective proteins and osmolytes. The review further evaluates the feasibility of breeding for drought tolerance alongside stable nutrient composition, considering genetic diversity, trait heritability, and genotype × environment interactions. Findings suggest that environmental effects strongly influence nutrient-related traits, limiting the effectiveness of selection based solely on genetics. While breeding exclusively for nutritional traits may yield modest gains, integrating drought-resilient genotypes with water-efficient agronomic practices and improved value-chain management offers a more robust pathway. Overall, this review highlights key drought adaptation mechanisms in *Amaranthus* and underscores its potential as a climate-resilient crop for sustainable and affordable leafy vegetable production in sub-Saharan Africa.

## 1 Introduction

African Indigenous Vegetables (AIVs) have potential to contribute to nutrition security across sub-Saharan Africa. They provide essential micronutrients, enhance dietary diversity, and in addition serve as important sources of household income. Beyond their nutritional value, these crops contribute to agro-biodiversity and strengthen the resilience of local food systems (Achigan-Dako et al., 2014). Among these vegetables, amaranth (*Amaranthus* spp.), often referred to as an *“opportunity crop”*, stands out as one of the most widely cultivated and consumed leafy vegetables in Africa and some other parts of the world (Sarker and Oba, 2018a). It is considered one of the most important African Indigenous Vegetables (AIVs) due to its dual use offering nutrient-rich leafy vegetable, protein and micronutrient-dense seeds (Vidal Torres et al., 2024).

Across sub-Saharan Africa, *Amaranthus* has been proposed as an underutilized crop with potential relevance for climate-stressed and low-input systems. Some species exhibit rapid early growth, heat tolerance and rich nutrient profile, however, agronomic performance and nutritional value are strongly dependent on species, genotype, and environmental conditions (Nyonje et al., 2025). Yield and quality remain sensitive to nutrient limitations, drought severity and timing, underscoring the need for context-specific evaluation rather than generalized claims of resilience. The leaves are notable sources of protein, iron, zinc, calcium, vitamins A and C, as well as secondary metabolites such as phenolics and flavonoids, which possess antioxidant properties, while antinutrients like oxalate and phytic acid may limit mineral bioavailability. Meanwhile, the seeds provide high-quality proteins and lipids, including squalene (Mukuwapasi et al., 2024). Both leafy and grain types thus make substantial contributions to food and nutrition security in the region. Beyond direct nutrition, amaranth’s bioactive components and processed flour are applied in commercial food, medicinal, and cosmetic sectors as functional ingredients with nutraceutical value (Jan et al., 2023).

Very high and very low humidity remains a persistent constraint to amaranth production and nutrient content across Africa. Increasing climate variability has intensified the frequency and severity of drought events, directly impairing physiological processes and nutrient accumulation in both leaves and grains (Netshimbupfe et al., 2023; Nyonje et al., 2025). Water deficits alter mineral uptake and affect the biosynthesis of key metabolites, including antioxidants and secondary compounds, with potential downstream impacts on nutritional quality and consumer acceptance (Sarker and Oba, 2018b; Nyonje et al., 2025). While certain antioxidants and osmolytes may accumulate to enhance stress tolerance, essential minerals such as K, Ca, and Fe often decline, resulting in reduced nutritional quality (Netshimbupfe et al., 2023). Consequently, drought is one of the most critical abiotic stresses limiting both yield and nutrient stability in *Amaranthus* spp.

The efficiency of modern breeding program is gauged by rate of genetic gain per unit time. One of the key innovations that have fasten the variety development is advancement made in genomic research for most key crops such as; wheat, maize, rice, beans and cassava (Varshney et al., 2019), which genomic resources have led to use of molecular breeding techniques to reduce breeding cycles and quicken delivery of improved varieties to farmers (Xu and Crouch, 2008). Unfortunately, AIVs that provide the much-needed food, nutrition and income security in Africa have had limited research investment to match the gain attained by staple crops. It’s imperative to develop modern tools such as genome-wide association studies (GWAS) and multi-trait selection to enhance both drought tolerance and nutritional quality. Yet, progress remains limited by insufficient genomic resources, underinvestment in research on indigenous crops, and fragmented breeding programs across African research institutions (Mukuwapasi et al., 2024). Given amaranth’s dual role as a food security crop and a source of micronutrients and bioactive compounds, developing drought-resilient varieties with stable nutrient accumulation has become a strategic research priority.

In this review, we examine the major breeding strategies applied to enhance drought resilience and nutrient stability in *Amaranthus* spp. from exploiting genetic diversity to participatory breeding approaches. We further highlight key advances, existing gaps, and opportunities for leveraging amaranth as a climate-smart, nutrient-rich African Indigenous Vegetable capable of strengthening resilient food systems in sub-Saharan Africa.

## 2 Methodology

This review was developed through a systematic and thematic examination of peer-reviewed publications, technical reports, and relevant grey literature addressing Amaranthus breeding, drought resilience, and nutrient accumulation. Literature searches were conducted across major scientific databases, including ScienceDirect, SpringerLink, Wiley Online Library, African Journals Online (AJOL), MDPI, Taylor & Francis, and Google Scholar. Search queries combined keywords such as “Amaranthus spp.”, “drought tolerance”, “nutrient accumulation”, “African Indigenous Vegetables”, “breeding strategies”, “biofortification”, “GWAS”, and “molecular markers in amaranth”. All retrieved publications were screened for relevance based on their contribution to understanding the effects of drought stress on the physiological, biochemical, and nutritional composition of Amaranthus, as well as on breeding approaches aimed at resilience and nutritional enhancement. Studies were included if they presented original research, reviews, or technical reports linking drought stress to nutrient composition or if they provided insights into breeding strategies, genomic resources, or molecular tools applicable to Amaranthus. Publications were excluded when they focused solely on agronomic performance without genetic or physiological perspectives, lacked nutritional data, or were written in languages other than English unless they offered critical contextual information.

The final selection of literature was synthesized thematically to capture key dimensions of *Amaranthus* research, including its origin and distribution, nutritional and health relevance, physiological and biochemical responses to drought, and breeding strategies for enhanced resilience and nutritional quality. This integrative approach enabled the identification of research gaps, methodological advances, and emerging directions that could inform future efforts to position *Amaranthus* as a climate-smart, nutrient-rich crop capable of strengthening food and nutrition security across sub-Saharan Africa.

### 2.1 Local names and symbolic meanings of *Amaranthus* spp. in some Africa nations

The genus *Amaranthus* consists of more than 70 species, ranging from pseudo-grain, leafy vegetables, to ornamental and weedy amaranth (Assad et al., 2017), that play a central role in traditional diets across Africa, the Middle East, and Asia. The plant is known by a wide variety of vernacular names that vary across ethnic groups and regions, reflecting linguistic diversity and the cultural meanings attached to it within different communities.

In East Africa, *Amaranthus* shows remarkable variation in naming and utilization. In Kenya, it ranks among the most consumed African Indigenous Vegetables and is cultivated as both a leafy green and grain crop. It is known as terere among the Kikuyu, *D*ododo among the Luo, and chepkarta among Kalenjin communities. The leaves are typically sautéed or boiled and served with ugali, while grain amaranth is increasingly valued for its nutraceutical potential and used to fortify maize flour (Abukutsa-Onyango, 2021). In Uganda and Rwanda, amaranth, commonly called Ddodo, is a staple leafy vegetable widely grown and marketed as a household relish, though its commercial potential remains underexploited (Ssepuuya et al., 2019). In Tanzania, known locally as mchicha, it is one of the most important vegetables in urban and peri-urban food systems and a key component of the traditional ugali–vegetable meal (Msuya et al., 2010). In Ethiopia, the crop is known as aleko, aluma, hemberxefa and tete, and is consumed mainly as a leafy vegetable, although its popularity remains geographically limited compared to vegetables such as kale (Alemayehu et al., 2015; Dinssa et al., 2019).

In West Africa, the linguistic and cultural diversity of *Amaranthus* also reflects its integration into local diets. In Benin, it is one of the most widely cultivated African Indigenous Vegetables, grown across agroecological zones for household consumption and market sales, and commonly referred to as fotètè in the Fon language (Achigan-Dako et al., 2014). In Togo, it is called fotètè *or* gboma-fotètè and remains a cornerstone of traditional cuisine despite limited scientific research on its nutritional variability and breeding potential (Dansi et al., 2008). In Ghana, it is known as aleefu among the Ewe and bεkontire in Akan-speaking areas and is among the most consumed leafy vegetables used in soups and stews (Abizari et al., 2017). In Nigeria, it has several local names including ẹ̀fọ́ tete among the Yoruba, alefu among the Hausa, and akwụkwọ anara ofe among the Igbo, illustrating its strong cultural presence in household meals and markets (Oloyede et al., 2021). In Côte d’Ivoire, it is also known as fotètè and cultivated mainly as a seasonal leafy vegetable but remains under-researched in agronomic and nutritional terms (Dansi et al., 2008).

In Southern Africa, *Amaranthus* is also widely consumed. In Zimbabwe, where it is called mowa, it is cultivated in home gardens and collected from the wild as a seasonal relish contributing to nutritional security (Moyo et al., 2015). In Zambia, it is known as bonongwe and is commonly boiled and eaten with nshima, the national staple (Nguni et al., 2011). The same name is used in Malawi, where both fresh and dried leaves are consumed, with dried leaves serving as an important dry-season food source (Chikowo et al., 2014). Despite its popularity, research on varietal diversity and improvement remains limited (Grubben and Denton, 2004). In South Africa, *Amaranthus* species are collectively referred to as morogo or imifino, terms used for a variety of wild and traditional leafy greens. The plant is widely consumed in rural communities for its nutritional and medicinal value but remains underutilized in formal food systems (Vorster et al., 2002; Mavengahama et al., 2013). Across Africa, these vernacular names and uses highlight the cultural embeddedness of *Amaranthus*, reflecting its nutritional, economic, and symbolic roles in African food cultures.

Traditional medical uses of *Amaranthus* across Africa also highlight its therapeutic value. Preparations from leaves, roots, stems, and ashes have been used to treat conditions such as constipation, fever, wounds, and anemia (Topwal, 2019). Pharmacological studies, particularly on *A. spinosus*, report antioxidant, hepatoprotective, antimicrobial, anti-inflammatory, antiviral, antimalarial, and immunostimulatory activities consistent with its ethnomedicinal applications (Kumar et al., 2014).

### 2.2 Amaranthus: a strategic crop for reducing food and nutrition insecurity

Although notable global progress has been made in reducing nutrition-related challenges, particularly in low-income countries (Bouis and Islam, 2011), many barriers continue to undermine these achievements. Climate change, poverty, inadequate nutrition education, and limited access to diverse, affordable foods remain major obstacles to food and nutrition security (Muthayya et al., 2013). Limited awareness of nutrient-dense foods often prevents individuals from making informed dietary choices. Vegetables, especially *Amaranthus*, are increasingly recognized for addressing hidden hunger and improving dietary quality. However, consumer perceptions and socio-cultural dynamics often marginalize traditional crops. Exotic vegetables such as kale and collard greens are often associated with higher social status and receive greater research and policy attention, yet remain less accessible to poor populations, while indigenous vegetables like *Amaranthus* are undervalued despite being more affordable (Achigan-Dako et al., 2014). Many consumers remain unaware that traditional vegetables including *Amaranthus* are rich in essential vitamins, minerals, and phytochemicals with nutraceutical potential (Ramdwar et al., 2017).

Despite global research and investment focusing on exotic crops, neglected traditional crops such as *Amaranthus* have received limited attention despite their contribution to food and nutrition security (NRC, 2006). *Amaranthus* is a nutrient-dense African Indigenous Vegetable whose nutritional benefits depend on genotype, environment, and processing. It is an affordable and sustainable option for combating diet-related diseases and nutrient deficiencies, particularly in staple-based diets. While not a true cereal like wheat, sorghum, or maize, *Amaranthus* is classified as a pseudo-cereal similar to buckwheat and quinoa (Achigan-Dako et al., 2014). Its seeds can be milled into flour for various food products, while the leaves are valued for their high nutritional content. Although sometimes labeled “pigweed,” *Amaranthus* is a versatile plant widely cultivated for food, feed, medicinal, ornamental, and industrial uses.

As a multipurpose crop, *Amaranthus* provides cereal-like grains and nutrient-rich leafy vegetables suitable for human and animal nutrition. Its composition includes high-quality protein, calcium, iron, vitamins A, C, and K, and B vitamins such as riboflavin, niacin, vitamin B6, and folate. Regular consumption has been associated with reducing malnutrition and micronutrient deficiencies (Qumbisa et al., 2020). However, the crop remains under-researched despite its nutritional and nutraceutical potential. The leafy vegetable and grains also have low calorie content, making them suitable for weight management. Although fresh leaves are widely available, processed amaranth products remain uncommon due to limited investment in value addition. Developing dried or powdered products could extend shelf life, improve year-round availability, and enhance food and nutrition security (Drzewiecki, 2001). Despite being drought-tolerant and highly nutritious, *Amaranthus* is gradually disappearing from some regions where it was once widely grown, highlighting its status as an underutilized crop. Given the persistent burden of malnutrition in many developing countries, greater research and investment are needed to harness *Amaranthus* as a sustainable solution for improving food and nutrition security.

### 2.3 Common species of *Amaranthus* grown for leaves and grains

Botanically, *Amaranthus* is a genus of herbaceous flowering plants with taxonomic and morphological characteristics that distinguish it from true cereals like wheat or rice. Amaranth is grown worldwide for its edible leaves and grains (Aderibigbe et al., 2022). There is no robust, current estimate of total global amaranth area in hectares in the scientific record; reported national figures range from a few thousand to several hundred thousand hectares, and global totals are not well documented. Among the species within the genus, three *A. cruentus*, *A. caudatus*, and *A. hypochondriacus* are recognized as the principal grain amaranths, although dual-type varieties of *A. caudatus* and *A. hypochondriacus* exist. *Amaranthus cruentus* and *Amaranthus hypochondriacus* are particularly notable for high protein content and richness in essential amino acids such as methionine and cysteine, as well as minerals and lipids (Aderibigbe et al., 2022). Both are consistently among the most nutrient-dense amaranths. The seeds are consumed cooked, as popped grain, or milled into flour and blended with other cereals to produce snacks and complementary foods.

**Figure 1:**
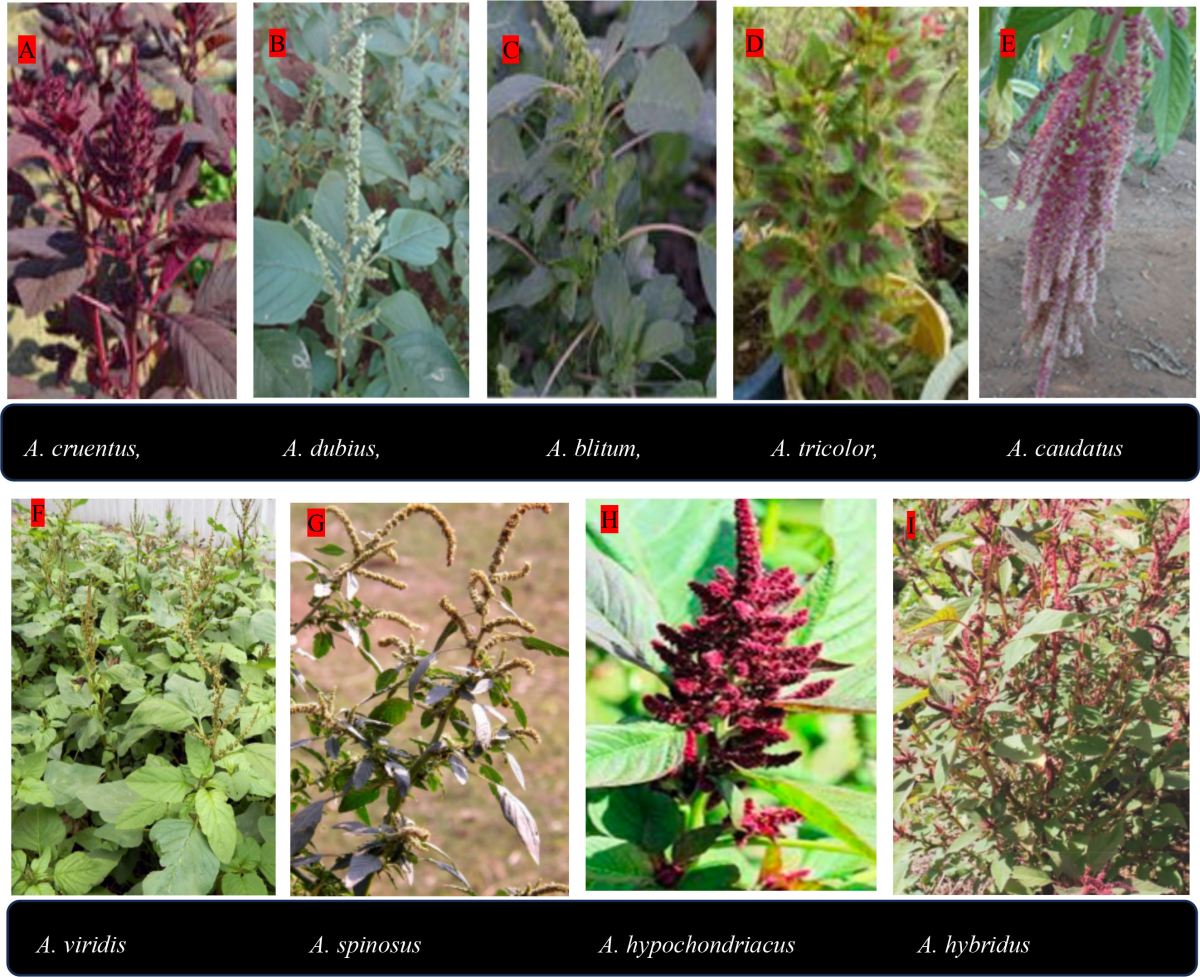
*Common amaranth species:* **(A)** Terminal inflorescence of *A. cruentus*; **(B)** Terminal inflorescence of *A. dubius*; **(C)** Apical and axillary inflorescences of *A. blitum*; **(D)** *A. tricolor*; **(E)**. Hanging inflorescence of *A. caudatus*; **(F)** Terminal inflorescence of *A. viridis*; **(G)**. Apical and axillary inflorescences of *A. spinosus*; **(H)** Dense inflorescence of *A. hypochondriacus*; **(I)**. Elongated inflorescence of *A. hybridus*. (Source: Bayón, 2022, licensed CC-BY-4.0).

The most widely cultivated leafy amaranths include *A. cruentus*, *A. dubius*, *A. blitum*, and *A. tricolor*. Among them, *A. cruentus* holds a unique position as a dual-purpose species cultivated for both leaf and grain production depending on market objectives (Aderibigbe et al., 2022). Several regionally important species are also used for ornamental, fodder, and medicinal purposes. For example, *A. dubius* (wild spinach in sub-Saharan Africa) is valued for its mild flavor and palatability and is consumed by both humans and livestock (Achigan-Dako et al., 2014). *A. viridis* is another widespread species that often grows wild but is also cultivated as a leafy vegetable and used in traditional medicine (Achigan-Dako et al., 2014). Morphologically, vegetable and grain amaranths differ in inflorescence structure and seed characteristics. Vegetable types generally have axillary glomerules or short spikes with flowers arising from leaf axils, three tepal lobes, and three stamens, and produce brownish-black seeds with an indeterminate growth habit (Achigan-Dako et al., 2014). Grain amaranths, in contrast, have apical inflorescences with complex branching, five stamens, and cymes of five tepal lobes. Their seeds range from white to cream or pinkish rather than dark brown or black and have a discoid shape with a folded flange region and a circumscissile utricle (Achigan-Dako et al., 2014). **Figure 1** shows amaranth inflorescences behavior.

Interestingly, the morphological boundaries between vegetable and grain species are often blurred. The young leaves of grain amaranths are edible and commonly used as pot herbs or side dishes. Some weedy species such as *A. spinosus* grow spontaneously in fallow lands and are harvested and consumed as vegetables. Seed characteristics therefore remain the most reliable trait for differentiating vegetable, grain, and weedy forms of *Amaranthus* (Achigan-Dako et al., 2014). Overall, the genus *Amaranthus* encompasses remarkable species diversity with overlapping uses and characteristics. Its versatility as a source of nutritious leaves, high-protein grains, traditional medicine, and livestock feed continues to position it as a valuable yet underexploited crop contributing to food and nutrition security across Africa and beyond. The most widely grown *Amaranthus* species, their synonyms and common names are described in **Table 1** below.

**Table 1:** Some of the most widely grown species and their common name.

Species	Synonyms (selected)	Common names	References
*Amaranthus blitum* L.	*A. lividus* L.; *A. oleraceus* L.	Slender amaranth, purple amaranth, pigweed, mchicha (Swahili)	Grubben and Denton, 2004; Royal Botanic Gardens Kew, 2024
*Amaranthus cruentus* L.	*A. hybridus* subsp. *cruentus* (L.) Thell.; *A. paniculatus* L.	African spinach, grain amaranth, mchicha	Grubben and Denton, 2004; National Center for Biotechnology Information (NCBI), 2024; Royal Botanic Gardens Kew, 2024
*Amaranthus caudatus* L.	*A. mantegazzianus*; *A. edulis*	Love-lies-bleeding, tassel amaranth	Iamonico, 2023; Royal Botanic Gardens Kew, 2024
*Amaranthus dubius* Mart. ex Thell.	*A. tristis* auct. non L.	Spleen amaranth, pigweed, mchicha	Grubben and Denton, 2004; Royal Botanic Gardens Kew, 2024
*Amaranthus hypochondriacus*L.	*A. hybridus* auct. non L.	Prince’s feather, grain amaranth	Grubben and Denton, 2004; Royal Botanic Gardens Kew, 2024
*Amaranthus spinosus* L.	—	Spiny amaranth, prickly pigweed	Grubben and Denton, 2004; Royal Botanic Gardens Kew, 2024
*Amaranthus thunbergii* Moq.	—	Wild amaranth, wild spinach	Grubben and Denton, 2004
*Amaranthus tricolor* L.	*A. gangeticus* L.; *A. tristis* L.	Chinese spinach, Joseph’s coat	Grubben and Denton, 2004; Royal Botanic Gardens Kew, 2024
*Amaranthus viridis* L.	*A. gracilis* Desf. ex Poir.	Green amaranth, slender amaranth, mchicha	Grubben and Denton, 2004; Royal Botanic Gardens Kew, 2024

### 2.4 Nutritional value and potential health benefits of *Amaranthus* leaves

Nutritionally, *Amaranthus* leaves provide protein and key micronutrients including zinc, calcium, magnesium, phosphorus, folate, potassium, iron, and vitamins A–C while remaining low in carbohydrates and total energy (Ramdwar et al., 2017). Compared with commonly consumed salad vegetables, *Amaranthus* often shows higher levels of iron, vitamin C, vitamin A (β-carotene), and calcium (Ramdwar et al., 2017). The crop’s rapid growth and short harvest cycle further strengthen its potential to support food and nutrition security (Singh, 2017). In addition to vitamins and minerals, *Amaranthus* leaves are abundant in antioxidants and other bioactive constituents that may help prevent or mitigate micronutrient deficiencies and diet-related non-communicable diseases (Jimoh et al., 2019). Some of the most common nutrients obtained in the crop are discussed below.

#### 2.4.1 Protein

Dark green *Amaranthus* leaves and especially the grain are notable sources of plant protein, including essential amino acids. The lysine content is comparatively high for a leafy vegetable (Schnetzler and Breene, 2018; Achigan-Dako et al., 2014). Protein-rich foods help regulate appetite and glycemia, which may support weight management (Ramdwar et al., 2017). As a gluten-free option, *Amaranthus* seed used in bread and pastries can benefit those with gluten intolerance. The grain provides 14–16 g protein per 100g while the leaves have around 3–6 g per 100 g edible portion, supplying a balanced amino-acid profile without gluten (Achigan-Dako et al., 2014). Proteins are critical for humans for growth, tissue repair, and enzymatic functions throughout the body (Jimoh et al., 2018).

#### 2.4.2 Fiber

*Amaranthus* leaves and grains offers both soluble and insoluble fiber, supporting gut health, weight management, and cardiovascular and metabolic risk reduction (Sarker and Oba, 2019). Its ease of digestion makes it suitable for convalescents; traditional uses include managing diarrhea and hemorrhages.

#### 2.4.3 Iron

Amaranth leaves contain significantly more Fe than grain. Typical iron concentrations in leaves range from ~7–15 mg Fe per 100 g fresh weight (≈60–120 mg Fe per 100 g dry weight)), while the grain contains ~7–9 mg Fe per 100 g dry weight (Achigan-Dako et al., 2014). The high iron content of amaranth supports erythropoiesis, prevents anemia and contributed to cellular metabolism (Kumar Maurya and Arya, 2018). Bioavailability improves with appropriate processing (e.g., brief blanching) and co-consumption of vitamin C–rich foods that enhance non-heme iron absorption (Yang and Tsou, 2006). Regular intake can contribute to preventing iron-deficiency anemia in resource-limited diets.

#### 2.4.4 Zinc

Amaranth leaves are also known for their high zinc content, which varies among species. Dinssa et al. (2020) reported that the zinc content in A. dubius is significantly higher than the content in A. cruentus and A. hypochondriacus based on evaluations conducted across two seasons in an on-station trial in Tanzania.

#### 2.4.5 Vitamins C

150–250 g of fresh amaranth leaves can supply the daily vitamin C requirement (Achigan-Dako et al., 2014). Vitamin C supports immune function, collagen synthesis, and iron absorption. *Amaranthus* also contains phenolics and flavonoids linked to antioxidant, anti-inflammatory, and potential anticarcinogenic actions, relevant to the prevention of atherosclerosis and other chronic diseases (Jimoh et al., 2019).

#### 2.4.6 Vitamin A

Leaves are rich in provitamin A carotenoids, with intake from typical portions approaching or exceeding daily requirements, supporting vision, immune competence, and fetal development (Achigan-Dako et al., 2014).

#### 2.4.7 Vitamin K

Green leaves of *Amaranthus* provide substantial phylloquinone (vitamin K1), essential for blood coagulation and bone health via osteocalcin carboxylation; vitamin K–mediated neuroprotective roles are also reported (Lee et al., 2017).

#### 2.4.8 Vitamin B-complex

Leaves supply a wide range of B-complex vitamins such as thiamin (B1), riboflavin (B2), niacin (B3), pyridoxine (B6), and folate (B9), with reports also noting cobalamin (B12) in some analyses (Randhawa et al., 2017). B-complex serve as coenzymes in energy metabolism of carbohydrates, fats, and proteins, support nervous-system function, and folate is central to one-carbon metabolism, nucleotide synthesis, and homocysteine regulation critical for maternal and neonatal health (Dai and Koh, 2015).

#### 2.4.9 Calcium

A typical cup of cooked leaves can contribute meaningfully to daily calcium intake, supporting bone mass and reducing risk of osteopenia/osteoporosis, particularly in low-dairy diets (Achigan-Dako et al., 2014).

#### 2.4.10 Potassium

*Amaranthus* is a useful source of potassium, an intracellular electrolyte essential for fluid balance, neuromuscular function, and blood pressure regulation (Palmer and Clegg, 2016). Given the high cost of many potassium-rich fruits and vegetables, *Amaranthus* offers an accessible option for low-income households (Randhawa et al., 2017).

### 2.5 Bioavailability of micronutrients in *Amaranthus* leaves

Several studies show that plant-based foods, including *Amaranthus* and other dark leafy vegetables, naturally contain varying concentrations of antinutritional compounds such as phytates, oxalates, tannins, and nitrates (Sanz-Penella et al., 2012). These compounds, termed antinutritional factors, are common in edible plants and can interfere with the absorption and utilization of essential nutrients, thereby reducing bioavailability (Jimoh et al., 2018). Antinutrients such as phytic acid, oxalates, proanthocyanidins, tannins, and certain dietary fibers can chelate minerals like iron, zinc, calcium, and magnesium, forming insoluble complexes that hinder gastrointestinal absorption (Funke, 2011). Despite these compounds, *Amaranthus* remains a nutrient-dense leafy vegetable rich in essential micronutrients and minerals.

The bioavailability of micronutrients in *Amaranthus* depends on food preparation methods, culinary combinations, and interactions among bioactive compounds. Processing techniques such as blanching, boiling, or fermentation can reduce antinutrient levels and enhance mineral availability, while excessive heating or prolonged cooking may cause nutrient losses (Jimoh et al., 2018). The absorption of fat-soluble compounds like β-carotene can also be improved by co-consumption with lipid-rich foods, while interactions of vitamin C, lutein, lycopene, and polyphenols enhance β-carotene stability and bioavailability (Jiménez-Aguilar and Grusak, 2017).

These findings suggest that dietary pairing and moderate processing can optimize nutrient retention and absorption in *Amaranthus*-based dishes. For example, combining *Amaranthus* leaves with vitamin C–rich foods such as tomatoes or citrus fruits, or cooking them lightly with oil, may improve assimilation of iron and provitamin A carotenoids. Thus, although antinutritional factors can limit nutrient uptake, appropriate culinary practices and food synergies can enhance the nutritional value of *Amaranthus* and its role in combating micronutrient deficiencies.

### 2.6 Barriers to adoption of amaranth – beyond nutritional promotion

Despite strong evidence demonstrating its nutritional, agronomic, and economic potential, amaranth remains underutilized in sub-Saharan Africa. A critical yet insufficiently explored issue is the lack of systematic research on barriers to adoption. Fragmented research investments and unsustainable donor-driven breeding initiatives have slowed progress, but a deeper problem lies in the imbalance between promotion and diagnosis. Previous research on African vegetables emphasized advocacy highlighting nutritional value and market opportunities rather than addressing structural, social, and institutional constraints preventing widespread uptake (Abukutsa-Onyango, 2019; Schreinemachers et al., 2021). As a result, although amaranth is often promoted as a “super leafy vegetable,” the reasons farmers have not fully integrated it into production systems remain poorly documented.

Limited public awareness of amaranth’s nutritional benefits is frequently cited as a barrier, but this alone does not explain farmers’ decision-making. Adoption is influenced by factors such as seed availability, labor requirements, agronomic reliability, market demand, cultural perceptions, and risk attitudes, but less by nutritional quality (Ambrose et al., 2020). For instance, even when consumers recognize the nutritional value of amaranth, inconsistent seed supply and lack of improved varieties can deter farmers from commercial production. Similarly, poorly developed market chains often limit profitable returns and discourage expansion (Schreinemachers et al., 2021). Short shelf life, limited post-harvest infrastructure, and inadequate extension services further reduce farmer confidence and uptake (Moyo et al., 2018).

Perceptions and cultural preferences also influence adoption. In many regions, amaranth is still viewed as a “poor man’s crop” or a weed, leading to undervaluation by producers and consumers (Maundu et al., 2009). Such attitudes require integrated behavioral and socioeconomic research rather than agronomic solutions alone. Moreover, health messaging about nutritional value does not necessarily create economic incentives. Farmers often prioritize traits such as drought tolerance, short growth cycle, ease of cultivation, and market access over micronutrient density (Gotor and Caracciolo, 2020). Breeding programs that focus solely on nutritional traits risk producing varieties that remain unused by farming communities.

Future research must therefore place adoption barriers at the center of breeding strategies and policy design. Systematic surveys, value-chain assessments, and participatory rural appraisal can help identify context-specific obstacles and guide development of farmer- and consumer-preferred varieties. Integrating farmers, traders, and consumers through participatory varietal selection and farmer-led evaluation can also align research priorities with real agricultural behavior. In conclusion, amaranth research must move beyond promotional narratives toward a diagnostic, demand-driven approach; without understanding why farmers have not adopted amaranth at scale, fragmented investment and limited impact will likely persist.

### 2.7 Impact of drought on nutrient profiles in amaranth

Drought stress significantly alters nutrient accumulation and partitioning in leafy vegetables, and evidence shows that amaranth, while highly resilient, experiences measurable biochemical and nutritional changes under water limitation (Sarker and Oba, 2020b). Moderate drought often induces osmotic adjustment and alters primary metabolism, increasing the density of some nutrients, whereas severe drought disrupts cellular integrity, mineral uptake, and secondary metabolite biosynthesis (Patel et al., 2022). Mild water stress can increase beneficial phytochemicals such as phenolics, flavonoids, and carotenoids that act as antioxidants (Sarker and Oba, 2018c). However, severe drought reverses these effects, reducing nutrient content and yield due to impaired photosynthesis and nutrient translocation. Micronutrient profiles respond differently depending on drought severity. Moderate drought may increase iron, zinc, and magnesium concentrations because reduced leaf expansion concentrates minerals in smaller biomass (Sarker and Oba, 2018c). It can also increase protein, ash, minerals (Fe, Zn, Ca, K), pigments, vitamins, polyphenols, and antioxidant capacity while carbohydrates and moisture decrease (Nkuna et al., 2025). Studies also show shifts in mineral composition of leaves and seeds under drought (Tetyannikov et al., 2022), while seed quality can remain stable even when yield declines (Pulvento et al., 2021). Conversely, severe drought often reduces uptake of nitrogen, potassium, and calcium due to impaired root activity and disrupted nutrient transport (Rosa et al., 2019). Although these changes affect nutrient balance, yield loss under severe drought often dominates, making concentration changes nutritionally less relevant for consumers. Drought also influences antinutritional compounds. Moderate drought can increase tannins, oxalates, phytates, and nitrates through stress-induced secondary metabolism (Ramakrishna and Ravishankar, 2011), while severe stress may further elevate oxalates and saponins, reducing nutritional value (Rosa et al., 2019). These responses appear genotype-specific, suggesting breeding for drought-tolerant and nutrient-stable varieties is possible but requires screening large germplasm collections under controlled drought conditions.

Drought timing and severity also influence nutrient responses. Early vegetative drought tends to affect mineral uptake, whereas later-stage drought more strongly influences secondary metabolites (Patel et al., 2022). Therefore, physiological stage, soil moisture dynamics, and genotype interactions must be considered. Future research should combine phenotyping of nutrient traits under drought with genome-wide association studies to identify loci controlling nutrient stability and support targeted breeding and agronomic recommendations.

### 2.8 Drought stress responses and adaptive mechanisms in plants with emphasis on *Amaranthus* spp.

**Figure 2:**
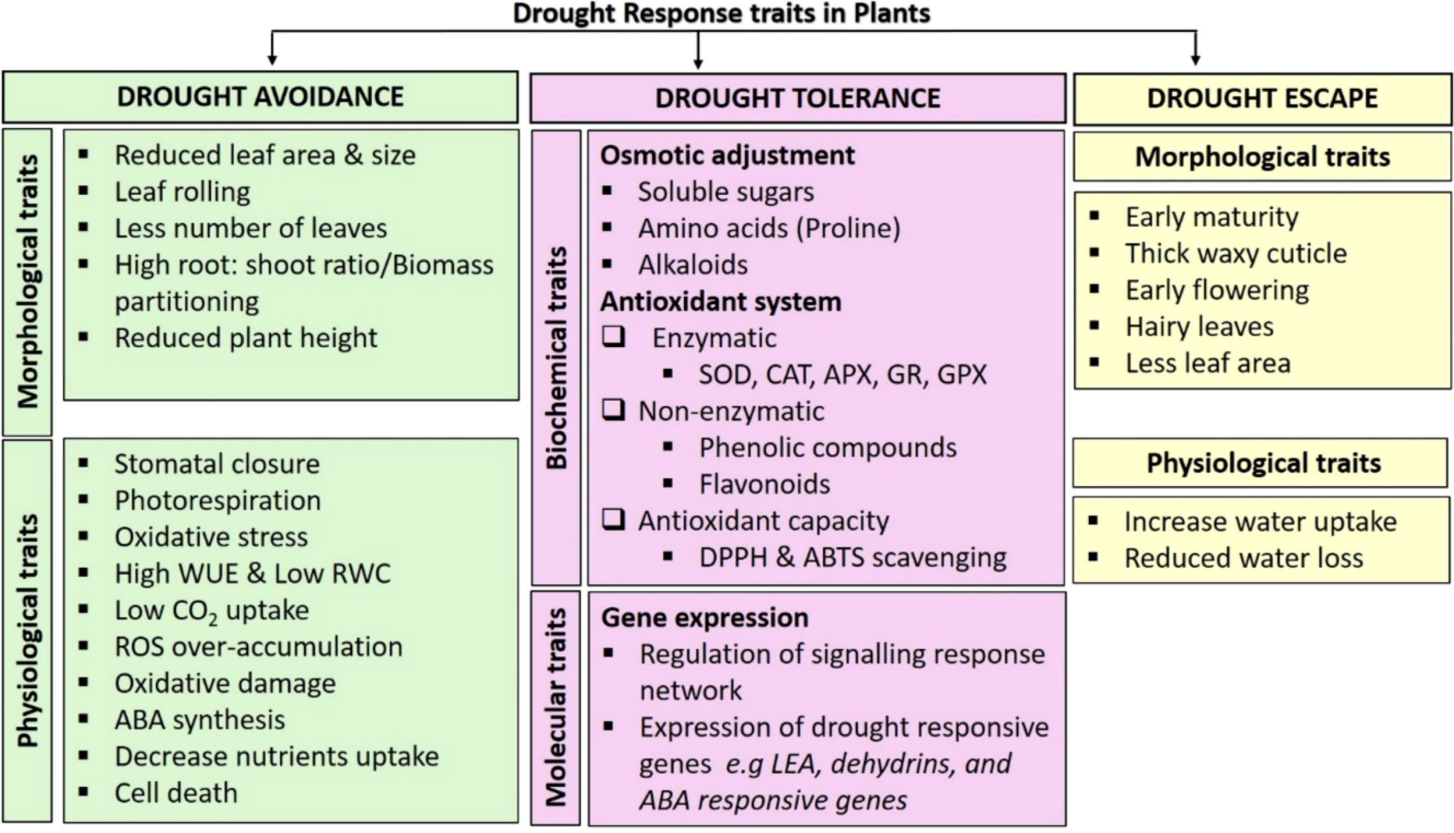
Plant response to drought stress changes at the morphological, physiological, biochemical, and molecular levels. ABA, abscisic acid; ABTS, 2,2′-azino-bis 3-ethylbenzthiazoline-6-sulfonic acid; APX, ascorbate peroxidase; CAT, catalase; CO_2_, carbon dioxide; DPPH, 2,2-diphenyl-1-picrylhydrazyl; GPX, glutathione peroxidase; GR, glutathione reductase; LEA, late embryogenesis abundant protein; ROS, reactive oxygen species; RWC, relative water content; SOD, superoxide dismutase; WUE, water use efficiency. (Source: Nkuna et al., 2025, licensed CC-BY-4.0).

Drought triggers water loss and a reduction in water potential, leading to decreased cell turgor (Hura et al., 2007). Prolonged drought conditions cause osmotic imbalance and ion-mediated oxidative stress, which adversely affect plant physio-chemical processes (Laxa et al., 2019). In response, plants activate a range of morphological, physiological, biochemical, and molecular mechanisms, as illustrated in **Figure 2**. These responses are broadly categorized into drought avoidance, escape, and tolerance. Drought avoidance involves morphological adaptations such as leaf curling, wax deposition on leaf surfaces, and reduced shoot–root ratio (Ahmad et al., 2023), as well as physiological adjustments like reduced relative water content (RWC), which promotes stomatal closure to limit water loss through transpiration (Pirasteh-Anosheh et al., 2016). Drought escape enables plants to complete their life cycle before the onset of severe drought (Kim et al., 2007), while drought tolerance is achieved through osmotic adjustment, activation of antioxidant metabolism, and expression of drought-responsive genes (Shavrukov et al., 2017).

Drought-induced changes disrupt photosystems I and II (PSI and PSII) in chloroplasts, leading to the generation of reactive oxygen species (ROS), including superoxide anion (O_2_•^-^), hydrogen peroxide (H_2_O_2_), and hydroxyl radicals (OH•). Excessive ROS accumulation causes oxidative damage to membranes, lipids, proteins, and nucleic acids, ultimately resulting in cell death (González-Rodríguez et al., 2019). To cope with stress, plants synthesize proteins and amino acids and accumulate minerals. They also regulate water relations through osmotic adjustment (OA), involving the buildup of low molecular weight solutes such as proline, glycine betaine, sugars, and free amino acids. This process helps maintain turgor pressure, sustain water content (Aranda-Rivera et al., 2022), and restore metabolic activity (Lisar et al., 2012; Zivcak et al., 2016). Additionally, increased activity of late embryogenesis abundant (LEA) proteins and ROS-detoxifying enzymes including superoxide dismutase (SOD), catalase (CAT), peroxidase (POD), glutathione reductase (GR), and enzymes of the ascorbate–glutathione cycle plays a key role in mitigating oxidative stress and enhancing tolerance (Ashraf, 2009). Stress-tolerant plants typically accumulate higher levels of osmolytes and non-enzymatic antioxidants and exhibit stronger expression of stress-responsive genes. Although sensitive crops activate similar mechanisms, they often fail to sustain growth and yield under severe stress (Laxa et al., 2019).

#### 2.8.1 Leafy vegetable amaranth species and their morphological, physiological, biochemical and molecular responses to drought stress

Leafy vegetable species of the genus *Amaranthus* represent an important component of food systems, particularly in tropical and subtropical regions, due to their availability during dry seasons when other leafy vegetables are scarce (Nkuna et al., 2025). These species are typically characterized by relatively short growth habits (up to ~1.5 m), succulent stems, broad and smooth leaves, and small auxiliary inflorescences (Ribeiro et al., 2017). Among the numerous species within the genus, *Amaranthus tricolor*, *Amaranthus hybridus*, and *Amaranthus dubius* are the most widely studied in relation to drought stress due to the availability of experimental data (Nkuna et al., 2025). Their responses to water deficit involve coordinated changes at morphological, physiological, biochemical, and molecular levels, reflecting complex adaptive strategies to environmental stress.

#### 2.8.2 Morphological responses

Drought stress markedly influences morphological development in *Amaranthus* spp., Moyo et al. (2022) demonstrated that polyethylene glycol (PEG 6000)-induced drought in *A. dubius* reduced final germination percentage, mean germination time, germination index, and germination rate index. In a comparative study, (Ribeiro et al., 2017) evaluated *A. tricolor* and *A. hybridus* under 20% (SDS), 50% (MDS), 80% (LDS), and 100% (control) total available water during vegetative growth. Under LDS, plants exhibited shorter internodes, increased lateral shoots, and smaller leaves, although leaf area remained unchanged in both species. Amma and Rajalakshmi (2023) similarly reported reduced plant height in *A. tricolor* compared to *A. dubius* under progressive drought. Biomass reduction under MDS (50% FC) and SDS (25% FC) has been widely documented (Sarker and Oba, 2018d), including significant declines in yield and biomass across 44 A*. tricolor* accessions (Jamalluddin et al., 2021) and a 43.18% reduction in plant height in *A. hybridus* under greenhouse conditions (Manyatsi et al., 2012). Notably, the *A. tricolor* cultivar “Hin Choi” exhibited faster recovery after rewatering, with improved leaf area development compared to *A. blitum* and *A. cruentus* (Liu and Stützel, 2004; Bai et al., 2021). Overall, reduced growth and biomass accumulation represent key morphological drought-response traits that help minimize water loss (Ribeiro et al., 2017).

#### 2.8.3 Physiological responses

Physiological responses in *Amaranthus* spp. are largely governed by plant water relations and photosynthetic performance. *A. dubius* maintained higher relative water content (RWC) under drought (85.66%) compared to *A. tricolor* (72.29%), whereas *A. hybridus* showed a 30% reduction in RWC after 10 days of water deprivation (Vargas-Ortiz et al., 2021). Compared to grain amaranth (*A. cruentus*), *A. dubius* exhibited a greater decline in RWC (43%), indicating higher water loss (Ferrarotto, 2003). Photosynthetic parameters such as chlorophyll fluorescence and gas exchange remain relatively stable under LDS (80% FC) but decline significantly under MDS (50% FC) and SDS (25% FC) (Emmanuel and Babalola, 2022.; Sarker and Oba. 2018d). For example, chlorophyll a and b decreased by ~50% under SDS (12.5% WHC) in *A. hybridus* (Asadollahzadeh et al., 2023). In contrast, Motyleva et al. (2021) reported a two-fold increase in chlorophyll and carotenoids in *A. tricolor* cv. “Valentina” under moderate/severe drought (30–40% soil moisture). Drought also reduced chlorophyll fluorescence parameters such as quantum efficiency, photochemical quenching, and electron transport rate in *A. hybridus* (Ribeiro et al., 2017). Membrane stability declined significantly, with chlorophyll stability index reduced by 70–75% in *A. dubius* and 50% in *A. tricolor*, and cell membrane stability decreasing by 55–70% under short-term water stress (Amma and Rajalakshmi, 2023). Although *A. hybridus* shows some drought tolerance (Liu and Stützel, 2004), it is generally less tolerant than grain *Amaranthus* spp. under SDS (González-Rodríguez et al., 2019). These findings suggest that efficient water use and biomass partitioning are key physiological mechanisms, though highly genotype-dependent.

#### 2.8.4 Biochemical responses

Drought stress induces notable biochemical changes in leafy *Amaranthus* spp. Under low LDS (80% FC), oxidative stress markers such as malondialdehyde (MDA), H_2_O_2_, electrolyte leakage (EL), proline, and non-enzymatic antioxidants (carotenoids, ascorbic acid, polyphenols, flavonoids, and total antioxidant capacity) show minimal changes but increase progressively under MDS (50% FC) and SDS (25% FC) (Sarker and Oba, 2018d). Osmotic adjustment (OA) is evident through increased accumulation of osmolytes, particularly proline in *A. hybridus* and *A. dubius* (Asadollahzadeh et al., 2023; Vargas-Ortiz et al., 2021), and soluble sugars in *A. hybridus* under severe stress (Vargas-Ortiz et al., 2021). Enhanced antioxidant capacity is also reported, including higher DPPH scavenging activity, total phenolic content (TPC), amino acids, reducing sugars, and ascorbic acid in *A. tricolor* (Chatti, 2017; Motyleva et al., 2021). In contrast, *A. dubius* shows high antioxidant activity (DPPH and ABTS) even under non-stress conditions (Aderibigbe et al., 2022). Enzymatic antioxidants such as superoxide dismutase (SOD), catalase (CAT), glutathione reductase (GR), monodehydroascorbate reductase (MDHAR), ascorbate peroxidase (APX), and dehydroascorbate reductase (DHAR) are strongly induced, particularly in drought-tolerant genotypes (e.g., VA13), indicating efficient ROS detoxification (Sarker and Oba, 2018d). Increased activities of SOD, CAT, and glutathione peroxidase (GPOX) have also been observed in *A. hybridus* under extreme drought (Aderibigbe et al., 2022). These findings confirm that antioxidant defense is a key biochemical mechanism of drought tolerance, often associated with lower ROS levels (H_2_O_2_, MDA, EL) and enhanced stress resilience (Gill and Tuteja, 2010; Sarker and Oba, 2018d; Zhou et al., 2019).

#### 2.8.5 Molecular responses

Molecular studies on drought responses in leafy vegetable amaranths remain relatively limited, although available genomic resources provide a foundation for future research. Chloroplast genome sequencing has been completed for several species, including *A. tricolor*, *A. hybridus*, and *A. dubius*, revealing genome sizes of approximately 150 kb, GC content of ~36.5%, and a conserved set of protein-coding, rRNA, and tRNA genes (Li et al., 2015). These genomic resources are valuable for phylogenetic studies and marker development. Genetic diversity analyses using molecular markers such as simple sequence repeats (SSRs), single nucleotide polymorphisms (SNPs), and the maturase K (*matK*) gene have demonstrated substantial variation within *A. tricolor* populations (Xu et al., 2021). High-throughput approaches such as double-digest restriction-site-associated DNA (ddRAD) sequencing have identified thousands of SNPs across global germplasm collections, highlighting significant genetic diversity and suggesting possible misclassification of some accessions (Hoshikawa et al., 2023). These studies also point to regions such as China and India as potential centers of origin for *A. tricolor*. Despite these advances, there is a notable gap in understanding the molecular mechanisms underlying drought tolerance in leafy amaranths. Comparative studies suggest that *A. hybridus* may be more sensitive to drought at the molecular level compared to grain amaranths (Mabhaudhi et al., 2019), but detailed gene expression and regulatory network analyses remain scarce. This gap presents an important opportunity for integrating genomics, transcriptomics, and metabolomics to elucidate drought-responsive pathways and identify candidate genes for breeding climate-resilient amaranth cultivars. The availability of fully sequenced genomes is fundamental for understanding species-level metabolic regulation driven by molecular and genetic responses (Kersey et al., 2020). In *Amaranthus hypochondriacus*, genome sequencing has revealed a genome size of approximately 466 Mb, a diploid chromosome number of 2n = 32, and the coding capacity for at least 24,829 proteins (Sunil et al., 2014). In comparison, *A. cruentus* and *A. caudatus* exhibit similar genomic architectures, with genome sizes of 370.9 Mb and 398 Mb, respectively, a haploid chromosome number of n = 17, and an estimated 25,477 protein-coding genes (Ma et al., 2021). These genomic resources have significantly advanced the understanding of stress adaptation mechanisms within the genus *Amaranthus*.

Proteomic studies have demonstrated that drought stress induces substantial changes in protein expression in *A. hypochondriacus*, particularly in root tissues. Differential expression analyses have identified the upregulation of key stress-responsive proteins, including chloroplast chaperonin 60 kDa subunits (Cpn60α and Cpn60β) and heat shock protein 70 (HSP70) under drought conditions (Huerta-Ocampo et al., 2009, 2011). These findings suggest that chloroplasts and mitochondria play central roles in drought adaptation in *A. hypochondriacus* (Rastogi and Shukla, 2013). The induction of these proteins is functionally significant, as they stabilize cellular proteins by preventing aggregation and facilitating the refolding of denatured proteins under both normal and stress conditions (Clerico et al., 2019). Additionally, *A. hypochondriacus* appears to activate coordinated regulatory networks involving growth-related proteins (such as tyrosine phosphatases), reactive oxygen species (ROS)-scavenging enzymes, glutathione S-transferases, heat shock proteins (HSPs), and RNA-binding proteins (RBPs) (Huerta-Ocampo et al., 2011) collectively supporting cellular homeostasis under water deficit conditions.

At the transcriptomic level, González-Rodríguez et al. (2019) reported the differential expression of genes involved in raffinose family oligosaccharide (RFO) biosynthesis in grain *Amaranthus* species under moderate drought stress (MDS) and severe drought stress (SDS). These include galactinol synthase genes (*AhGolS1* and *AhGolS2*), raffinose synthase (*AhRafS*), and stachyose synthase (*AhStaS*). RFOs play important roles in protecting embryos from desiccation and also function as signaling molecules during stress responses such as wounding and pathogen attack (Sengupta et al., 2015). Under SDS, *AhGolS1*and *AhRafS* were strongly induced in leaves across all examined species, correlating with increased raffinose accumulation. In contrast, *AhGolS* and *AhStaS* transcripts showed higher expression in roots than in leaves in *A. hypochondriacus* and *A. cruentus*, suggesting tissue-specific regulatory roles. Conversely, genes associated with trehalose biosynthesis and degradation, including class I and II trehalose phosphate synthase genes, were downregulated in *A. hypochondriacus* and *A. caudatus* under drought stress (González-Rodríguez et al., 2019). Given the central role of abscisic acid (ABA) in regulating plant water balance through stomatal control, the expression of ABA-responsive genes is critical for drought response characterization (Cechin et al., 2022). Huerta-Ocampo et al. reported elevated expression of ABA signaling-related genes, including *AhDREB2A* (dehydration-responsive transcription factor), *AhABI5*(bZIP transcription factor), *AhRAB18* (ABA-responsive gene), and *AhLEA14* (late embryogenesis abundant protein gene) in roots of *A. hypochondriacus* and *A. cruentus* under drought stress (González-Rodríguez et al., 2019). However, *AhDREB2A* showed stronger expression in leaves under moderate drought conditions, indicating organ-specific regulation (González-Rodríguez et al., 2019).

**Figure 3:**
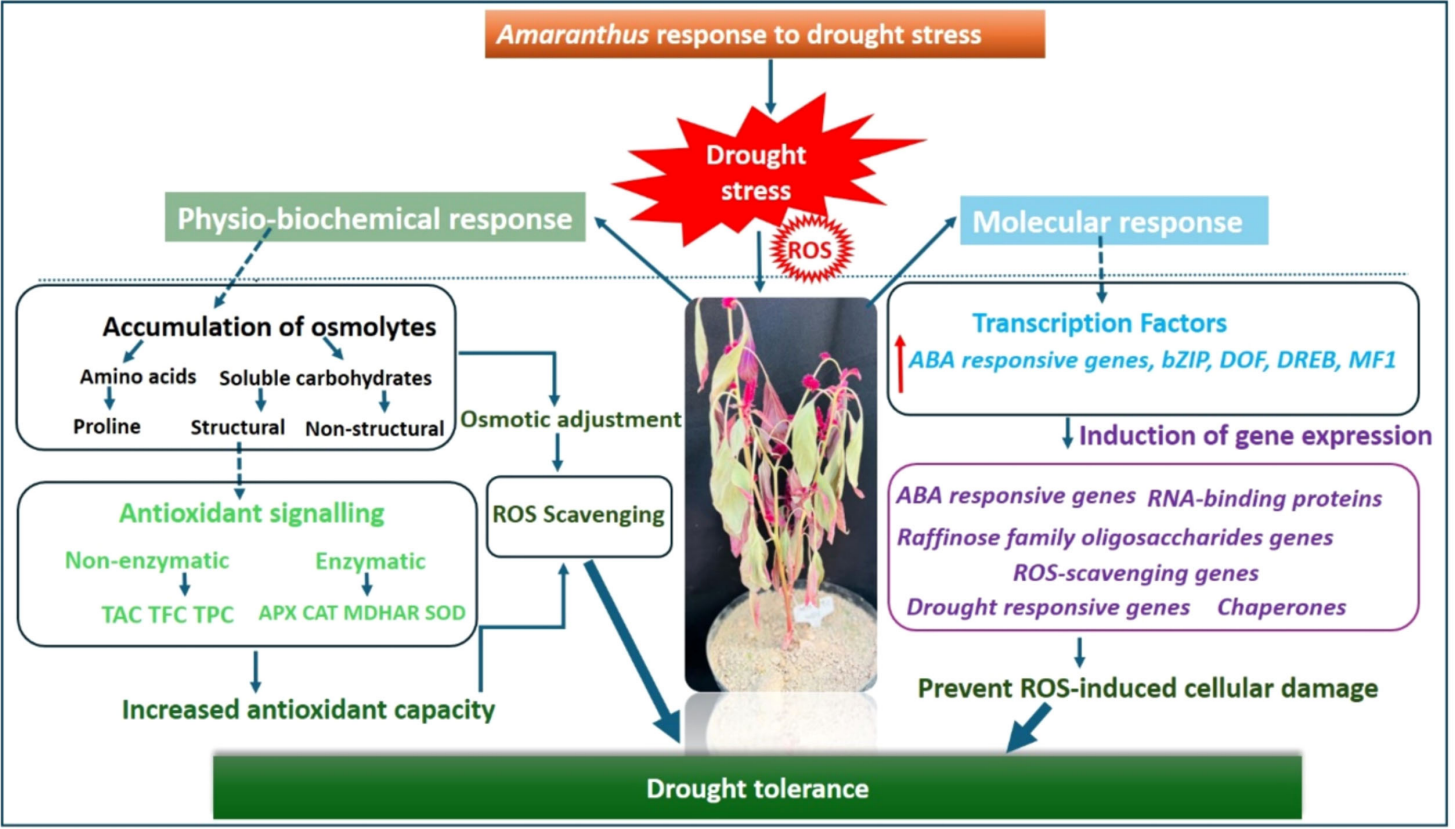
Mechanism of amaranthus drought stress tolerance. (Source: Nkuna et al., 2025, licensed CC-BY-4.0).

Further, Casique-Arroyo et al. (2014) investigated drought-induced regulation of betacyanin biosynthetic genes in three *A. hypochondriacus* genotypes (Nutrisol, India Red, and India Green). The study reported downregulation of *AhDODA-2*and cytochrome P450 gene (*AhCYP76*) in leaves of the Nutrisol genotype. In contrast, drought-stressed stems of Nutrisol and India Green exhibited upregulation of *AhDODA-1*, betanidin 5-O-glucosyltransferase (*AhB5-GT*), cyclo-DOPA 5-O-glucosyltransferase (*AhcDOPA5-GT*), and *AhB5-GI*, indicating tissue-specific activation of pigment biosynthesis pathways (Casique-Arroyo et al., 2014). Moreover, functional validation studies by Palmeros-Suárez et al. demonstrated that overexpression of the *A. hypochondriacus* Nuclear Factor Y (NF-Y) transcription factor enhances drought tolerance in *Arabidopsis thaliana*, a drought-sensitive model species (Palmeros-Suárez et al., 2015). Collectively, these findings suggest that improved osmotic adjustment, activation of ABA signaling pathways, and enhanced stress-responsive gene expression constitute key molecular and biochemical strategies underlying drought tolerance in grain *Amaranthus* species, particularly *A. hypochondriacus* (**Figure 3**).

### 2.9 Breeding amaranth for nutritional quality, antinutrient reduction, and drought tolerance: do genetic gains justify the investment?

Efforts to breed amaranth for improved nutrient content and reduced antinutrient levels need careful evaluation before major investments are made. Although amaranth is widely recognized as a nutrient-dense African vegetable rich in vitamins, minerals, and bioactive compounds, nutrient concentrations are often strongly influenced by environmental conditions rather than genetics (Achigan-Dako et al., 2014). Drought, soil fertility, harvesting stage, and postharvest handling frequently explain variability in protein, phenolics, carotenoids, and mineral composition more than genotype alone (Muchoki et al., 2020). Genetic control of micronutrients, phenolics, or antioxidant activity is moderate at best, while antinutrient content is highly environmentally regulated and plastic under abiotic stress (Sarker and Oba, 2020a). Differences in nutrient concentration among accessions appear relatively small and often overlap due to environmental noise, suggesting that breeding for nutritional quality alone may produce only marginal improvements unless environmental management is addressed (Chivenge et al., 2015).

Breeding for drought tolerance presents a more feasible target but requires clear trait definition and controlled phenotyping, as demonstrated in crops such as maize (CIMMYT, 2016). In amaranth, drought tolerance involves osmotic adjustment and root architecture (Osei-Yeboah et al., 2020), yet larger root systems may divert assimilates away from leaf biomass that consumers value. Therefore, breeders must evaluate root traits carefully and maintain balanced root-to-shoot carbon allocation (Pal et al., 2017). Given these challenges, an integrated strategy is needed. Screening genebank collections under controlled environments is essential to quantify real genetic variation in nutrient and antinutrient traits. Drought-focused breeding should target physiological traits with proven heritability such as transpiration efficiency, chlorophyll retention, or recovery ability. Nutritional improvement can also be achieved through postharvest technologies, agronomic practices, irrigation management, and soil health. Breeding goals must consider farmer adoption drivers including yield stability, taste, market preferences, seed availability, cooking quality, and pest resistance (Taylor et al., 2021).

In summary, breeding amaranth for higher nutrient content and lower antinutrient levels remains possible, but genetic gains are likely to be small because these traits depend heavily on the environment. Breeding for drought tolerance and agronomic performance appears more justified and farmer-relevant. A multidisciplinary strategy combining genetic resources, improved phenotyping, and socio-economic understanding offers the most practical pathway for advancing amaranth improvement and adoption.

### 2.10 Enabling drought-responsive amaranth breeding: germplasm, phenotyping, and crossing technologies

Advances in amaranth (*Amaranthus* spp.) breeding for high nutrient content and water stress tolerance will depend on effective exploitation of available genetic diversity and the development of reliable traits for selection under drought stress. Although amaranth is recognized as a climate-resilient leafy vegetable, breeding for improved nutrient quality and drought tolerance has been slow because collections of genetic resources are relatively small and underutilized. Public genebanks such as the USDA-ARS, World Vegetable Center (WorldVeg), and national seed banks across sub-Saharan Africa maintain hundreds to thousands of amaranth accessions, yet structured access, germplasm characterization, and formal pre-breeding activities remain limited (Sarker and Oba, 2020b). Digital genebank search tools and standardized passport data available at a few genebanks, including USDA-ARS and WorldVeg, provide an essential foundation for capturing diversity and identifying drought-related traits and nutrient stability under water limitation.

Phenotyping strategies for drought-influenced metabolite changes remain a major bottleneck in amaranth improvement. Traditional phenotyping relies on destructive chemical assays to quantify minerals, phenolics, flavonoids, betalains, proline, and antioxidant capacity under drought treatments (Ramakrishna and Ravishankar, 2011). However, these techniques are labor-intensive, expensive, and sensitive to environmental variation. Streamlined approaches such as high-throughput spectrophotometry, portable fluorometric sensors, and near-infrared reflectance spectroscopy (NIRS) allow rapid screening of large populations for metabolite changes (Sarker and Oba, 2018c; Vazquez-León et al., 2021). Phenotyping pipelines should integrate drought imposition protocols, trait prioritization, and stage-specific sampling because metabolite responses vary with growth stage, tissue type, and severity of water deficit. Linking these phenotypes with molecular markers through association mapping or genomic selection can improve prediction of metabolite responses under drought.

A major barrier to amaranth breeding is the technical difficulty of making controlled crosses due to small flowers, complex floral structures, variable pollen viability, and partial reproductive barriers among species (Stetter et al., 2016). These constraints limit the ability to combine traits such as drought tolerance, high nutrient content, and low antinutrient levels. Recent techniques including flower bagging, reduced pollen fertility of the female parent, staggered pollination timing, male sterility systems, embryo rescue, and greenhouse isolation protocols have improved crossing success and hybrid recovery (Wu et al., 2022). In addition, mutation breeding generates genetic variability for selecting stress-resilient traits, with mutant *Amaranthus tricolor* lines showing improved growth and survival under water-limited conditions (Slabbert et al., 2013; Nkuna et al., 2024).

Solving crossing barriers is critical for future genetic improvement because hybrid populations enable recombination, selection, and trait introgression from wild relatives with superior drought tolerance and metabolite stability (Mlakar et al., 2016). When combined with genebank diversity screening and genomic tools such as SNP markers, breeders can identify parent lines with favorable metabolite responses and incorporate these traits into locally adapted cultivars. Ultimately, progress in amaranth breeding depends on access to diverse germplasm, scalable phenotyping, and improved crossing methods, supported by skilled breeders, sustained funding, and growing demand for improved seed. Without these improvements, breeding for drought-influenced metabolite traits will remain slow and fragmented.

### 2.11 Sustainable production strategies for affordable leafy vegetables with amaranth as a model crop

Sustainable production of affordable leafy vegetables in sub-Saharan Africa requires an integrated approach that addresses climate variability, production costs, nutritional security, and farmer profitability. One strategy is the diversification of Traditional African vegetables (TAVs) with complementary agronomic traits to support continuous harvesting and year-round supply. Amaranth (*Amaranthus* spp.), African nightshade (*Solanum scabrum*), spider plant (*Cleome gynandra*), and cowpea leaves (*Vigna unguiculata*) differ in water use efficiency, heat tolerance, pest susceptibility, and seasonal adaptability, allowing farmers to maintain production across dry, wet, and transitional seasons (Abukutsa-Onyango, 2019). Mixed and sequential cropping of multiple AV species spreads climatic and market risks, enhances soil cover, reduces pest pressure, improves dietary diversity, and helps address micronutrient deficiencies (Maseko et al., 2021).

Agroecological practices represent another pathway for sustainable AV production. Conservation agriculture combining minimum tillage, residue retention, and crop diversification helps maintain soil moisture, improve soil organic matter, and stabilize yields under water scarcity (Muriuki et al., 2021). Amaranth is well suited to these systems. Organic mulching reduces evapotranspiration, moderates soil temperature, increases fertilizer use efficiency, and can extend harvesting intervals for leafy amaranth under smallholder conditions (Wainaina et al., 2020). These water-saving practices are particularly important in peri-urban vegetable production where water access is limited and irrigation costs are high. Because leafy vegetables have short growth cycles, integrating mulching with staggered planting enables continuous marketing and reduces pressure on freshwater resources. Deficit irrigation is another management approach that improves efficient use of scarce water resources without jeopardizing marketable leaf yield. Mild to moderate water reduction can maintain or enhance amaranth leaf quality while lowering irrigation costs, making it economically attractive for smallholder farmers (Omondi et al., 2024). Deficit irrigation can also be combined with greywater use, water harvesting, and localized irrigation methods such as drip and pitcher irrigation to improve water-use efficiency in resource-poor environments (Müller-Mahn et al., 2022). When integrated with soil-improving practices, these systems sustain productivity and reduce input dependency, supporting climate adaptation and environmental sustainability.

Overall, integrating multiple AV species with agroecological water-saving practices provides a framework to make leafy vegetable production more resilient, productive, and affordable. Amaranth stands out due to its rapid growth, wide genetic diversity, and adaptability to low-input, water-efficient production methods. Future research should test combinations of TAV species under different agroecological regimes using participatory approaches with farmers to identify effective management packages that enhance nutrition, food security, and sustainable vegetable production in drought-prone regions of Africa.

## 3 Conclusion

Amaranth is a climate-resilient crop with strong potential to enhance food and nutrition security in sub-Saharan Africa. Drought stress significantly affects nutrient dynamics: moderate stress may increase certain minerals and bioactive compounds, while severe water limitation reduces growth, yield, and nutritional stability, reflecting complex genotype × environment interactions. Its C4 photosynthesis confers high water-use efficiency, making it a promising candidate for drought-resilient breeding without compromising nutrition (Vidal Torres et al., 2024). Molecular breeding, genomics, and metabolomics reveal genetic diversity, stress-responsive genes, and metabolic pathways for nutrient retention under drought (Sunil et al., 2022). While breeding for nutritional quality is important, greater impact may come from prioritizing drought tolerance, yield stability, and farmer-preferred traits. Integrating breeding with improved agronomy, efficient water management, and stronger value chains is critical. Furthermore, comprehensive proteomics, transcriptomics, and metabolomics studies on vegetable amaranths are necessary to uncover drought-responsive traits in these crops. Therefore, multidisciplinary approach combining genetic improvement, agroecological systems, and market-oriented development offers the most effective pathway to position amaranth as a sustainable, nutrient-rich crop supporting resilient food systems in Africa.
